# Behaviour influences thermoregulation of boreal moose during the warm season

**DOI:** 10.1093/conphys/coaa130

**Published:** 2021-01-08

**Authors:** Daniel P Thompson, John A Crouse, Perry S Barboza, Miles O Spathelf, Andrew M Herberg, Stephanie D Parker, Max A Morris

**Affiliations:** Alaska Department of Fish and Game, Kenai Moose Research Center, 43961 Kalifornsky Beach Road, Suite B, AK 99669, Soldotna, USA; Department of Wildlife and Fisheries Sciences, Texas A&M University, TAMU 2258 Bldg. 1537, 534 John Kimbrough Blvd., College Station, TX 77843, USA; Alaska Department of Fish and Game, Kenai Moose Research Center, 43961 Kalifornsky Beach Road, Suite B, AK 99669, Soldotna, USA; Department of Wildlife and Fisheries Sciences, Texas A&M University, TAMU 2258 Bldg. 1537, 534 John Kimbrough Blvd., College Station, TX 77843, USA; Alaska Department of Fish and Game, 333 Raspberry Road, Anchorage, AK 99518, USA; Minnesota Department of Natural Resources, 1601 Minnesota Drive, Brainerd, MN 56401, USA; Department of Wildlife and Fisheries Sciences, Texas A&M University, TAMU 2258 Bldg. 1537, 534 John Kimbrough Blvd., College Station, TX 77843, USA; Department of Geography, Texas A&M University, Eller Oceanography and Meteorology Building, TAMU Bldg. 0443, 797 Lamar St, College Station, TX 77843, USA

**Keywords:** Activity, Alaska, *Alces alces*, behaviour, moose, rumen temperature, thermoregulation

## Abstract

Management of large herbivores depends on providing habitats for forage supply and refuge from risks of temperature, predation and disease. Moose (*Alces alces*) accumulate body energy and nutrient stores during summer, while reducing the impact of warm temperatures through physiological and behavioural thermoregulation. Building on the animal indicator concept, we used rumen temperature sensors and GPS collars on captive moose (*n* = 6) kept in large natural enclosures to evaluate how behaviour and habitat selection influence the rate of change in rumen temperature during the growing season on the Kenai Peninsula, Alaska, USA. We compared movement and habitat selection of individual females during tolerance days (daily amplitude in rumen temperature was ≥1.2°C in 24 h) with those of control days (daily amplitude in rumen temperature was < 1.2°C) before and after the tolerance day. Moose moved more during tolerance days (172 m • h^−1^; 95% confidence intervals (CI)  = 149–191 m • h^−1^) than on control days (151 m • h^−1^; 95% CI = 128–173 m • h^−1^). The rate of change in rumen temperature (°C • h^−1^) declined with low to moderate movement rates that were probably associated with foraging in all habitats. Movement only increased the rate of change in rumen temperature at high activity (~ > 500 m • h^−1^). Additionally, the relationship between rate of change in rumen temperature and movement rate was different during tolerance and control days in open meadow and wetland habitats. In all habitats except wetlands, the rate of change in rumen temperature increased while resting, which probably is a result of diet-induced thermogenesis. Our study demonstrates that the behavioural choices of moose on the landscape are associated with the rate of change in rumen temperature and their ability to thermoregulate. Wildlife managers must consider high-value habitats where wildlife can employ both behavioural and physiological mechanisms to tolerate warm ambient conditions in a landscape of forage, predators and pests.

## Teaser

Cold-adapted moose use behavioural responses to augment their ability to maintain body temperature while foraging in a warm environment. During the warm season, the hourly rate of change in moose rumen temperature is associated with movement rates that also vary by habitat.

## Introduction

The ability for wildlife to obtain, utilize and conserve energy and protein for maintenance, growth and reproduction varies based on the species and the landscape they inhabit (Barboza *et al.*, 2009). The value of the landscape for an animal depends on trade-offs between food supplies, environmental demands and risks of injury or death (e.g. movement, food quality and quantity, environmental conditions and predation risk; Laundré *et al.*, 2001; Wilson *et al.*, 2012; Long *et al.*, 2016; Gallagher *et al.*, 2017). Fluctuations in environmental temperatures can influence an animal’s movement and food intake (McFarland *et al.*, 2014; Camp *et al.*, 2018; Haase *et al.*, 2019) and activate physiological, behavioural and endocrine responses for thermoregulation (Silanikove, 2000; Cain III *et al.*, 2006; Terrien *et al.*, 2011). Animals may alter their behaviour to reduce their exposure to unfavourable environmental conditions (e.g. burrowing; Fick *et al.*, 2009) or to alleviate a current thermoregulatory demand (e.g. drinking; Brain and Mitchell, 1999). Behavioural responses to warm environmental temperatures in ungulates include solar orientation of the body (Maloney *et al.*, 2005b; Hetem *et al.*, 2011; Lease *et al.*, 2014), decreasing or shifting activity patterns (Maloney *et al.*, 2005a; Shrestha *et al.*, 2014; Boyers *et al.*, 2019) and selecting habitats on the landscape that provide access to shade, water or wind (Valeix *et al.*, 2008; Hetem *et al.*, 2012; Wiemers *et al.*, 2014; McCann *et al.*, 2016). The actual thermoregulatory benefit to the animal from these behavioural responses can only be assumed without simultaneously monitoring the subsequent effect on body temperature.

Continuous body temperature measurements have been collected in wild ungulates through biologging technology (Fuller *et al.*, 2005; Signer *et al.*, 2010; Rey *et al.*, 2016; Thompson *et al.*, 2018). Following the animal indicator concept (Franzmann, 1983), continuous body temperature measurements could be used as a physiological indicator for an animal’s relationship with their environment (Thompson *et al.*, 2020). Prior studies have documented core body temperature in conjunction with activity levels in ungulates (Turbill *et al.*, 2011; Riek *et al.*, 2017; Arnold *et al.*, 2018; Boyers *et al.*, 2019) but did not evaluate how activity or behaviour is associated with body temperature. Burrow use of Arctic ground squirrels (*Spermophilus parryii*) influenced core body temperature (Long *et al.*, 2005), while core body temperature was influenced by swimming, drinking and sand bathing in African primates (Brain and Mitchell, 1999; McFarland *et al.*, 2019). The effects of a warming climate on behaviour have been documented in northern ungulates (Long *et al.*, 2014; Brivio *et al.*, 2019; Jennewein *et al.*, 2020); however, the behavioural implications of these choices for maintaining body temperature have not been tested. We studied how behaviour is associated with body temperature in moose (*Alces alces*), a cold-adapted ungulate, to understand the effects of thermal trade-offs for a large animal in a warming climate.

Moose are generally solitary animals that rely on high forage intakes during the short, warm season to accumulate body energy and protein reserves (Schwartz *et al.*, 1984; Shively *et al.*, 2019), with ~ 75% of activity associated with foraging during this time frame (Ballenberghe and Miquelle, 1990; Herberg, 2017). As environmental temperatures increase, activity levels of moose decrease and may shift to a nocturnal activity pattern (Demarchi and Bunnell, 1995; Dussault *et al.*, 2004; Street *et al.*, 2015; Montgomery *et al.*, 2019; Jennewein *et al.*, 2020). Additionally, moose respond to warm ambient temperatures at both the landscape level by selecting forested habitat types for thermal cover and wetland areas (Renecker and Hudson, 1990; Dussault *et al.*, 2004; Broders *et al.*, 2012; van Beest *et al.*, 2012; Street *et al.*, 2015; Wattles *et al.*, 2018; Alston *et al.*, 2020; Jennewein *et al.*, 2020) and at a microhabitat level by selecting resting sites that increase heat loss (McCann *et al.*, 2016; Olson *et al.*, 2016). We recently validated devices to continually log core temperature in moose (Herberg *et al.*, 2018; Thompson *et al.*, 2018) and subsequently established daily and seasonal core body temperature patterns for moose (Thompson *et al.*, 2019). Combining continuous body temperature measurements of moose, with movement and habitat selection may allow us to evaluate the relationship between the individual animal and their daily trade-offs on the landscape.

Following the animal indicator concept, we used internal body temperature sensors and GPS collars on captive moose to evaluate if behavioural choices are associated with the rate of change in rumen temperature during the growing season on the Kenai Peninsula, Alaska. Here, we expand on our prior research in which we use the individual daily amplitude in core body temperature to determine when moose tolerate fluctuations in daily core body temperature above established seasonal values (Thompson *et al.*, 2020). We identify individual ‘tolerance days’ as a day in which an individual moose rumen temperature had a daily amplitude ≥1.2°C (tolerance day = heat response day in Thompson et al. 2020). Therefore, we consider a control day as the day prior and day after a ‘tolerance’ day in which that individual moose’s rumen temperature had a daily amplitude < 1.2°C. We hypothesize that (i) the daily patterns in hourly rate of change in rumen temperature, movement rates and drinking for moose would differ between a tolerance day and a control day. During a tolerance day, we predict moose would exhibit an increased rate of change in rumen temperature compared to a control day. Additionally, on tolerance days, we predict moose would reduce movement rates and increase drinking events in the late afternoon and early evening as body temperature, ambient air temperature and vapour pressure typically rise (Thompson *et al.*, 2020). Furthermore, we hypothesize that (ii) the rate of change in moose rumen temperature is dependent on movement rates and habitat selection and would differ between tolerance and control days. We predict that moose will decrease movement rates and select habitats (e.g. wetlands) that would decrease the rate of change rumen temperature on tolerance days. We also evaluated habitat selection by moose to determine if activity within habitats and resting site selection varied between tolerance and control days. We hypothesize that (iii) moose activity status (resting and active) would change within habitats during tolerance and control days. We predict that the time of day in which moose would select typically cool habitats (e.g. wetlands) would change during a tolerance day, with a higher probability of selection during resting bouts. Finally, we hypothesize that (iv) moose would select resting sites based on microhabitat attributes, which would aid in thermoregulation. We predict moose would select resting sites during tolerance days that contained microhabitats, which would be elevated (i.e. access to wind) and have higher canopy cover than the immediate surrounding habitat.

## Materials and methods

### Study site

We studied free-ranging (i.e. kept in a natural conditions) captive moose on the Kenai Peninsula, Alaska, USA. Moose were held in two 2.6 km^2^ outdoor enclosures at the Kenai Moose Research Center, operated by the Alaska Department of Fish and Game on the Kenai National Wildlife Refuge. We used an orthorectified aerial photo (Nikon D800; resolution, 7360 × 4912; focal length, 24 mm; pixel size, 4.88 × 4.88 μm; flight, 24 April 2015; elevation, 629 m; U.S. Fish and Wildlife Service, Kenai National Wildlife Refuge) to manually digitize vegetation polygons within each enclosure (ArcGIS 10.6; ESRI, Redland, CA, USA). We classified vegetation polygons as early-seral boreal forest (2–5 years post-disturbance, open canopy), mid-seral boreal forest (25 years post-disturbance), old growth boreal forest (65+ years post-disturbance), black spruce forest, wetland (kettle ponds and/or sphagnum peat bogs with areas of standing water) and open meadow. One enclosure also contained a 0.16 km^2^ lake. Vegetation classifications were sampled for tree density and to identify understory species (Supplementary Data S1).

### Environmental conditions

Ambient air temperature (°C), precipitation (mm), wind speed (m • s^−1^), solar radiation (W • m^−2^) and relative humidity were recorded every 5 min at a National Oceanic and Atmospheric Administration (NOAA) US Climate Reference Network weather station located at the Kenai Moose Research Center (Diamond *et al.*, 2013). Relative humidity and ambient air temperature were used to calculate dew point temperature (°C) and vapour pressure (hPa; Alduchov and Eskridge, 1996). We recorded ambient air temperature every 5 min in the six habitat types with Thermochron iButtons (accuracy, 0.5°C; Model DS1922L#F50; Maxim Integrated, San Jose, CA, USA) placed in yellow iButton key ring mounts (Model DS9093AY; Maxim Integrated). We suspended iButtons (*n* = 30; five in each habitat) from an i-bolt 120 cm off the ground and 10 cm from the north side of trees in forested habitats or from a steel T-post in open habitats. iButtons were strategically placed within each habitat type to collect representative data and to cover the study area. Additionally, we suspended five iButtons on the NOAA weather station to validate the iButtons. We used new iButtons at the start of the study that were calibrated by the manufacturer.

### Animal handling

We chemically immobilized adult, non-reproductive female moose (≥2 years old; *n* = 6) in December 2014 using procedures detailed in Herberg *et al.* (2018). While moose were immobilized, we fitted an Iridium global positioning system (GPS) collar (0.5 h fix rate; GPS Plus-7; Vectronic Aerospace GmbH; Berlin, Germany) and deployed a mortality implant transmitter as a rumen bolus (accuracy, 0.1°C; Vectronic Aerospace GmbH, Berlin, Germany; Minicucci *et al.*, 2018). Mortality implant transmitters recorded reticulorumen (hereafter rumen) temperature approximately every 5 min that we previously validated with these same moose (Herberg *et al.*, 2018). Rumen temperature was transmitted and stored on the GPS collar. All procedures for animal care, handling and experimentation were approved by the Animal Care and Use Committee, Alaska Department of Fish and Game, Division of Wildlife Conservation (protocol no. 09-29 and protocol no. 2014-17) and Texas A&M AgriLife Research Agricultural Animal Care and Use Committee (AUP# 2016-008A).

### Data analysis

We evaluated data collected during the warm season from 1 May 2015 to 31 August 2015. Rumen temperature was recorded approximately every 5 min but did not always correspond to the 5 min demarcations for all other measurements; therefore, we linearly interpolated rumen temperature to align with the rest of the dataset (Herberg *et al.*, 2018). Additionally, we linearly interpolated rumen temperature for any missing values (<2% of rumen temperature records). We identified drinking events (*n* = 794) as dips in rumen temperature below 37.5°C and the subsequent 40 min as rumen temperature returned to within one standard deviation of the initial rumen temperature (Herberg *et al.*, 2018). Additionally, we flagged any drop in rumen temperature >0.2°C within 5 min as a drinking event (Arnold *et al.*, 2018). We created a binary variable with 0 as no drinking event and 1 as a drinking event to censor the temperature record. To establish directional changes in body temperature, we linearly interpolated the rumen temperature for data that was flagged as a drinking event. We determined the rate of change in rumen temperature (°C • h^-1)^ by subtracting the rumen temperature 0.5 h prior from the current rumen temperature. We identified tolerance days for individual moose as days in which their rumen temperature, after removing drinking events, had a daily amplitude ≥1.2°C (Thompson *et al.*, 2020). For statistical comparison, we created a categorical variable for daily thermoregulatory response with two levels: tolerance day and control day based on individual daily amplitude in rumen temperature.

We removed moose locations if the GPS horizontal dilution of precision was ≥10 m (0.4% of GPS points) and replaced those locations by linear interpolation of the coordinates from acceptable locations before and after. We calculated movement rate (m • h^−1^) for each successive 0.5 h location. We transformed the continuous variable of movement rate with a natural log and then used a spline fit (seven-knot spline based on percentiles for large sample sizes; Harrell, 2001) for the model assessing if movement rate is associated with the rate of change in rumen temperature. To delineate potential resting sites for moose, we flagged all GPS locations with a movement rate ≤15 m • 0.5 h^−1^ based on the estimated GPS error from other studies (Lowe, 2009; McCann *et al.*, 2016). For habitat analyses, we created the categorical variable activity status with two categories: resting (movement rate <15 m • 0.5 h^−1^) and active (movement rate ≥15 m • 0.5 h^−1^). We spatially joined moose locations from the GPS collars with vegetation polygons (ArcGIS Pro 2.4.2; ESRI, Redland, CA, USA). We reclassified any moose GPS location in the lake to wetland. Additionally, any GPS location that fell outside of an enclosure was reclassified as the habitat closest to it within the enclosure (e.g. a moose was next to the enclosure fence and the GPS location was outside of the enclosure). We created binary variables for each habitat type to analyse probability of use.

We used light detection and ranging data (LIDAR; 1.2 m resolution; 2008; Kenai Peninsula Borough, Geographic Information Systems) to assess microhabitats associated with topography (digital elevation model) and canopy cover (digital surface model) around each moose GPS location. Using the package exactextractr (Baston, 2019) in R (R Core Team, 2018), we determined the mean value and raster pixel count in a 5 m and 25 m radius around each GPS location for both the digital elevation model and the digital surface model. We spatially joined the elevation value from the digital elevation model with each moose GPS location. To determine if the GPS location was above or below the surrounding area, we standardized the metric by subtracting the value for the mean elevation of the 5 m radius around the elevation of the GPS location. We used the digital surface model to estimate canopy cover surrounding each location in the forested habitats. We limited digital surface model data to values ≥1.5 m (i.e. above the height of a bedded moose) and assigned a value of 0.0 m to all early-seral vegetation polygons that were early-seral boreal forest as a result of habitat improvements after the LIDAR data were collected. We determined percent canopy cover for each GPS location by dividing the area within either the 5 m or 25 m radius that had a digital surface model value greater than 1.5 m, by the total area of the 5 m or 25 m radius from the digital elevation model, respectively.

We analysed data using programs in STATA version 15.0 (StataCorp LP, College Station, Texas, USA). We used simple linear regression to establish a relationship between ambient air temperature recorded from the NOAA weather station and iButtons located at the weather station. To evaluate the dependent variable of ambient temperature recorded with iButtons within each habitat, we used mixed model regression with the independent categorical variables of habitat type (six categories), time of day (0.5 h time periods starting at 0:00) and the interaction of habitat type and time of day. Using mixed model regression, we evaluated if the dependent variables (rate of change in rumen temperature; movement rate) were associated with the independent categorical variables of daily thermoregulatory response (control day vs. tolerance day), time of day and the interaction between daily thermoregulatory response and time of day. Similarly, we used mixed-effects logistic regression to determine if the probability of a drinking event was associated with the independent categorical variables of daily thermoregulatory response, time of day and their interaction. To evaluate if the dependent variable rate of change in rumen temperature was affected by movement rate and habitat selection, we used mixed model regression with the independent categorical variables for habitat type and daily thermoregulatory response, the two-way interaction of the categorical variables and the continuous variable for movement rate. Based on the results of this analysis, we further evaluated if movement rate and daily thermoregulatory response was associated with the change in rumen temperature in each specific habitat with mixed model regression. To determine the probability of moose selecting each habitat, we used mixed-effect logistic regression with the independent categorical variables daily thermoregulatory response, activity status (active and resting), time of day and all two- and three-way interactions of the independent variables. For microhabitat selection, we used mixed model regression for the three dependent variables (elevation of resting site; percent canopy cover of 5 m and 25 m radius) to determine if they were associated with the independent categorical variables daily thermoregulatory response, habitat type and the interaction of the independent variables. All mixed model regressions and mixed-effect logistic regressions included individual as a random effect. We reduced the effects of heteroscedacity and non-normal distributions with a robust sandwich estimator (Rabe-Hesketh and Skrondal, 2010). We examined linear fixed effects with a Wald test, and model explanatory variables were compared with zero using a *z*-test at *P* < 0.05. We then estimated marginal means (mean ± 95% confidence interval) to determine significant differences in the levels for each categorical variable. We performed model selection on all mixed and logistic regression models for physiological and behavioural responses in moose using Akaike’s information criterion, adjusted for small sample sizes (AIC*c*). We selected the simplest model with the lowest AIC*c* within two AIC*c* units of the top model (Burnham and Anderson, 2002).

## Results

### Environmental conditions

Early summer was cool and dry with daily low values of ambient air temperature (lowest daily minimum, −3.8°C; Supplementary Data S2) and vapour pressure (lowest daily minimum, 3.8 hPa; Supplementary Data S2) occurring in May. Daily mean ambient air temperature (12.4°C; range 5.0–19.8°C), vapour pressure (9.8 hPa, range 4.7–14.2 hPa) and solar radiation (191 W • m^−2^, range 49–341 W • m^−2^) all increased into the middle of summer, with the highest daily values of ambient air temperature (28.5°C; Supplementary Data S2) and solar radiation (1028 W • m^−2^; Supplementary Data S2) occurring in June, and peak vapour pressure in July (18.0 hPa; Supplementary Data S2). Cooler temperatures in late summer were accompanied by low vapour pressure and increasing daily precipitation (daily range, 0.0–12.4 mm; Supplementary Data S2) and wind (daily mean, 1.1 m • s^−1^; Supplementary Data S2).

**Figure 1:**
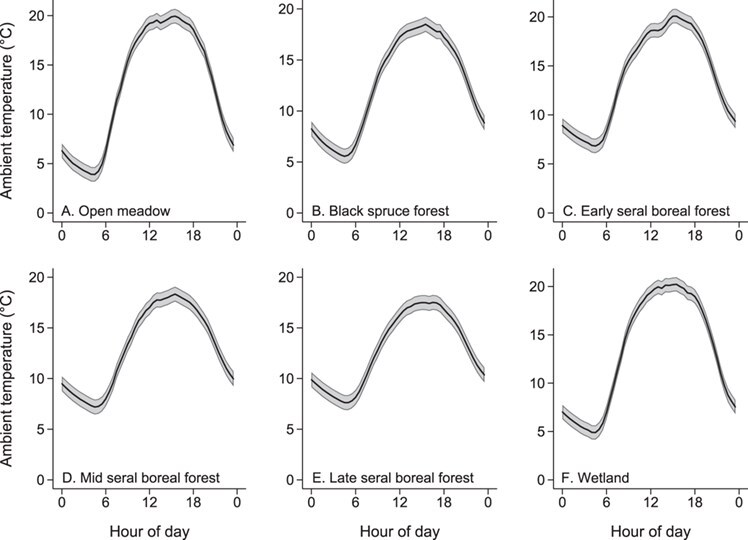
Patterns of daily ambient air temperature (°C) in six different habitat types (*n* = 30) during May through August 2015 at the Kenai Moose Research Center, Kenai Peninsula, Alaska, USA. Predicted values with 95% confidence intervals from mixed-effect model regression against time of day (0.5 h time periods starting at 0:00).

Ambient air temperature recorded with iButtons suspended on the weather station was similar to ambient air temperature recorded by the NOAA weather station sensor (*y* = 1.15*x − 0.93; *F*_1, 164 395_ > 99999.0; *P* < 0.001; *r^2^* = 0.96). Daily ambient air temperature patterns differed by habitats (Wald $\chi^2$ = 48991.7, *P* < 0.001); daily variation in open meadow and wetlands were greater than those of mid- and late-seral boreal forest (Fig. 1).

### Rumen temperature and behaviour

**Figure 2:**
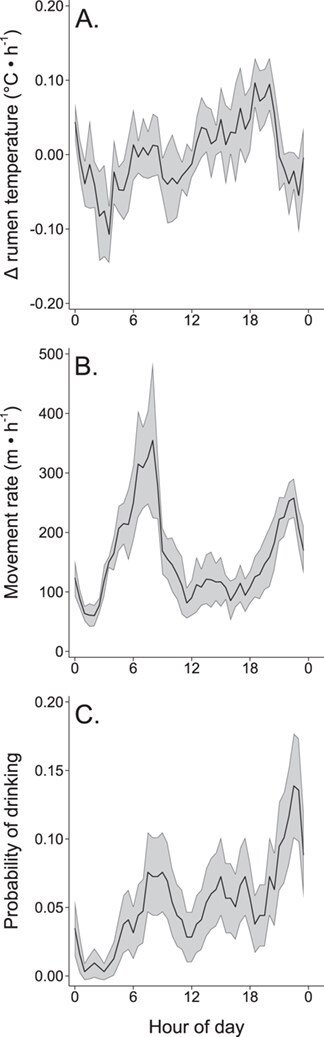
Daily patterns of (A) rate of change in rumen temperature (°C • h^−1^), (B) movement rate (m • h^−1^) and (C) probability of drinking for captive adult female moose (*n* = 6) at the Kenai Moose Research Center, Kenai Peninsula, Alaska, USA from May through August 2015. Predicted values with 95% confidence intervals from (A, B) mixed-effect regression and (C) mixed-effect logistic model regression against time of day (0.5 h time periods starting at 0:00). Daily sunrise (04:34–06:56) and sunset (21:13–23:39) varied over the summer.

We flagged 139 individual tolerance days (19% of all individual moose days) when the daily amplitude in moose rumen temperature was ≥1.2°C. Tolerance days occurred throughout the summer (67 out of 123 days; Supplementary Data S2). When considering time of day and the daily thermoregulatory response, the top model for the rate of change in rumen temperature (*n* = 15 216) only included the variable time of day (Wald $\chi^2$ = 66.1, *P* < 0.001; Supplementary Data S3) with a decreasing rate of change in rumen temperature in the early morning (Fig. 2A; maximum rate of decrease at 03:30 h; −0.10°C • h^−1^; 95% CI = −0.08–−0.14 °C • h^−1^) and increasing rate of change in the evening (Fig. 2A; maximum rate of increase at 18:30 h; 0.10°C • h^−1^; 95% CI = 0.06–0.12°C • h^−1^). Similarly, movement rates (*n* = 15 216; Wald $\chi^2$ = 456.2, *P* < 0.001; Supplementary Data S3) were highest during the morning (Fig. 2B) and greater on tolerance days (172 m • h^−1^; 95% *CI* = 149–191 m • h^−1^) than on control days (151 m • h^−1^; 95% CI = 128–173 m • h^−1^). Time of day was the only variable in the top model for the probability of drinking (Wald $\chi^2$ = 222.5, *P* < 0.001; Supplementary Data S3), which indicated the greatest probability of drinking in the evening (highest probability of drinking at 22:30 h; 0.14; 95% CI = 0.10–0.18; Fig. 2C).

**Figure 3:**
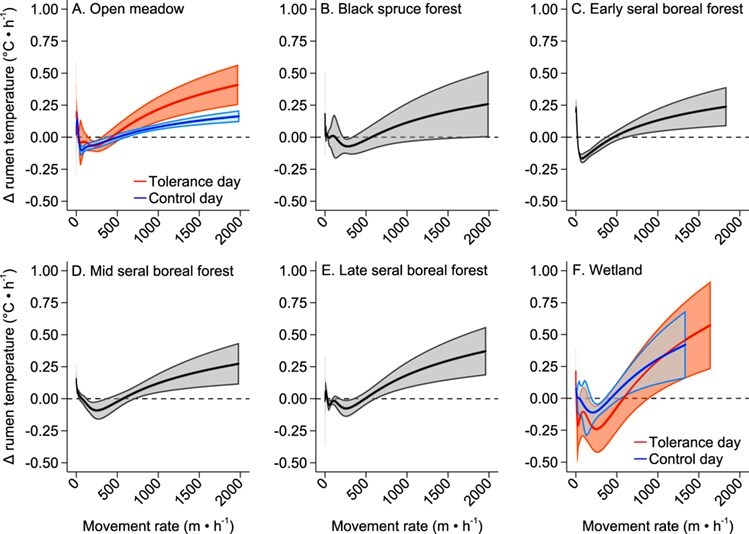
Rate of change in rumen temperature (°C • h^−1^) by movement rate (m • h^−1^) and daily thermoregulatory response within each habitat type (A–F) for captive adult female moose (*n* = 6) at the Kenai Moose Research Center, Kenai Peninsula, Alaska, USA from May through August 2015. Tolerance days were those days when individual moose had a daily amplitude in body temperature ≥1.2°C. Control days were the day prior and the day after a tolerance day. Predicted values with 95% confidence intervals from mixed-effect model regression.

**Figure 4:**
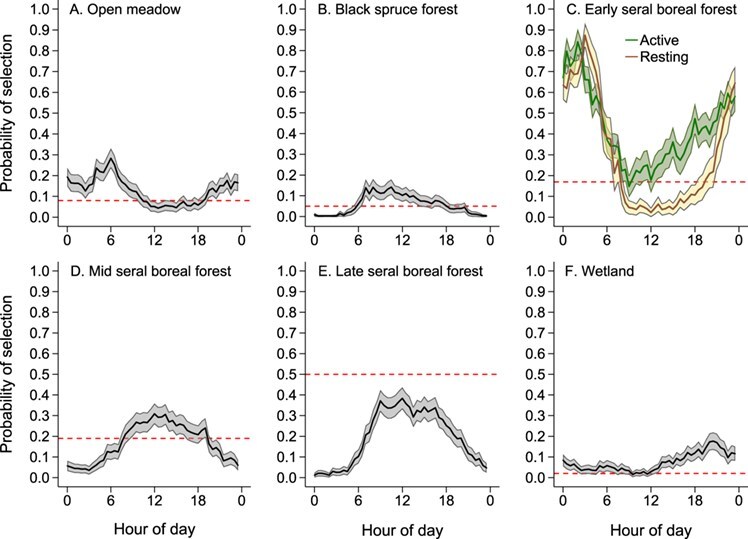
Probability of selection of each habitat type (A–F) by captive adult female moose (*n* = 6) at the Kenai Moose Research Center on the Kenai Peninsula, Alaska, USA from May through August 2015. Predicted values with 95% confidence intervals from mixed-effect logistic model regression against time of day (0.5 h time periods starting at 0:00) after selecting the best model describing probability of habitat selection for each habitat. Only the top model for early-seral boreal forest (C) included the interaction term between activity status and time of day. Horizontal red dashed line indicates the proportion of the total area each habitat encompassed (e.g. late-seral boreal forest comprised 0.5 or 50% of all vegetation within the study area).

For the model that assessed if habitat selection and movement is associated with the rate of change in rumen temperature, the top model that best described the rate of change in rumen temperature included the categorical variables for habitat type, daily thermoregulatory response, the interaction between habitat type and daily thermoregulatory response and the continuous variable movement rate (Wald $\chi^2$ = 9800000.0, *P* < 0.001; Supplementary Data S4). The rate of change in rumen temperature was different for tolerance and control days only in open meadow and wetland habitat types (Supplementary Data S5); however, although statistically significant, these small differences are less than the accuracy of the MIT rumen sensor. Evaluating the relationship between movement rate and rate of change in rumen temperature by each specific habitat, a different response curve between tolerance and control days was only observed in open meadow and wetland habitats (Fig. 3; *z* < 2.49, *P* < 0.013) compared to the forested habitats. Additionally, the rate of change in rumen temperature varied by movement rate; the rate of change in rumen temperature increased when movement rate was low (e.g. resting) in all habitats except on tolerance days in wetlands (Fig. 3E), while the rate of change in rumen temperature increased in all habitats when movement rate rose above ~ > 500 m • h^−1^ (Fig. 4). Decreases in the rate of change in rumen temperature occurred in all habitats with moderate movement rates ~ < 500 m • h^−1^ (Fig. 4).

### Habitat selection

The probability of moose selecting open meadow (Wald $\chi^2$ = 727.3, *P* < 0.001; Supplementary Data S6) included variables for activity status and time of day. Moose selected open meadow habitat more during the morning and less during mid-day (Fig. 4A), while the probability for selecting open meadow habitat was almost twice as high for resting (0.16, 95% CI = 0.14–0.19) than when active (0.09; 95% CI = 0.07–0.10). The probability of moose selecting black spruce forest (Wald $\chi^2$ = 514.1, *P* < 0.001; Supplementary Data S6) included the variables for activity status, daily thermoregulatory response and time of day. Moose selected black spruce forest in late morning but had very little use during the night (Fig. 4B). Additionally, the probability of moose selecting black spruce forest was higher when active (0.08, 95% CI = 0.04–0.12) than at rest (0.04; 95% CI = 0.02–0.06), while the probability of moose selecting black spruce forest was marginally higher during tolerance days (0.06, 95% CI = 0.03–0.09) compared to control days (0.05, 95% CI = 0.02–0.07). The probability of moose selecting early-seral boreal forest included the variables for daily thermoregulatory response and the interaction between activity status and time of day (Wald $\chi^2$ = 2671.0, *P* < 0.001; Supplementary Data S6). The probability of moose selecting early-seral boreal forest was lower on tolerance days (0.35, 95% CI = 0.33–0.37) than on control days (0.39, 95% CI = 0.37–0.41). Furthermore, the probability of moose selecting early-seral boreal forest was greater during early morning than during mid-day, with higher use while active during mid-day compared to resting (Fig. 4C). The probability of moose selecting mid-seral boreal forest (Wald $\chi^2$ = 878.9, *P* < 0.001; Supplementary Data S6) and late-seral boreal forest (Wald $\chi^2$ = 1286.5, *P* < 0.001; Supplementary Data S6) included the variables for activity status and time of day. Moose selected mid- and late-seral boreal forest during mid-day, with lower selection in early morning (Fig. 4D and E). Similarly, the probability of moose selecting both mid- and late-seral boreal forest was higher when active (mid-seral forest = 0.19, 95% CI = 0.16–0.22; late-seral forest = 0.23, 95% CI = 0.20–0.27) than when at rest (mid-seral forest = 0.14, 95% CI = 0.12–0.17; late-seral forest = 0.18, 95% CI = 0.15–0.21). The probability of moose selecting wetlands included the variables for daily thermoregulatory response, activity status and time of day (Wald $\chi^2$ = 475.3, *P* < 0.001; Supplementary Data S6). Moose selected wetlands with higher probability during late evening (Fig. 4F) and used wetlands more while active (0.10, 95% CI = 0.05–0.14) than resting (0.05, 95% CI = 0.03–0.07). Furthermore, moose selected wetlands with a slightly higher probability during tolerance days (0.08, 95% CI = 0.04–0.11) than during control days (0.06, 95% CI = 0.03–0.10).

### Microhabitat characteristics of resting sites

Moose selected resting sites for elevation based on habitat type (Wald $\chi^2$ = 2500000.0, *P* < 0.001; Supplementary Data S7) by selecting lower areas in wetlands than the surrounding area, while selecting elevated resting sites in all forested habitats (Supplementary Data S8). Additionally, moose selected resting sites for canopy cover based on habitat type within a 5 m (Wald $\chi^2$ = 114.3, *P* < 0.00; Supplementary Data S7) and 25 m radius (Wald $\chi^2$ = 58.46, *P* < 0.001; Supplementary Data S7) around the GPS location. Moose chose resting sites with canopy cover that was higher than the surrounding area in all forested habitats (5 m radius cover > 25 m radius cover; Supplementary Data S8). Furthermore, cover at resting sites in late-seral forest was greater than those in mid-seral forest and black spruce forest (Supplementary Data S8).

## Discussion

Building on the animal indicator concept, our study reveals some of the intricacies between moose physiology and behaviour as these animals navigate the landscape. Non-reproductive female moose in this study exhibited diurnal patterns in the rate of change in rumen temperature, movement rates and drinking during the warm season. Movement rates were greatest in the morning with selection for early-seral forest where forage is abundant. The rate of change in rumen temperature increased at rest when animals typically ruminate and also when movement rates were rapid; however, moderate movement rates were associated with decreasing rate of change in rumen temperatures, which declined rapidly in early-seral forest and during tolerance days in wetlands. During tolerance days, we documented different response curves for the rate of change in moose rumen temperature associated with movement rate when they were in open meadows and in wetlands. We did not detect any shift in the daily pattern for the probability of drinking or the rate of change in rumen temperature in moose during a tolerance day versus a control day. We did detect a difference in moose movement rate during a tolerance day; however, average movement rates increased on tolerance days, which was contrary to our initial hypothesis. In African elephants (*Loxodonta africana*), movement rate increased with ambient temperature, while using open habitat types, and as they approached water sources (Thaker *et al.*, 2019). Increased movement rates during tolerance days in moose could be a result of direct, quick movements to habitats that provide thermoregulatory relief while reducing the time they remained in open habitats during the day. Furthermore, increased movement rates in this study may also be related to insect avoidance (Renecker and Hudson, 1990); however, we could not detect this type of behaviour response with the data we collected. We also observed a higher rate of increase in the change in rumen temperature when movement rates were high (~ > 500 m • h^−1^). When planning habitat enhancements or evaluating the effect of natural disturbances, managers should consider the size and shape of habitat patches created on the landscape (Collins and Schwartz, 1998; Miner, 2000; Wattles and DeStefano, 2013). Large blocks of contiguous vegetation patches may increase the distance moose need to travel to access adequate forage or other resources that incur a thermoregulatory cost to the animal.

Similar to other studies on moose, we observed peaks in movement rates during crepuscular periods (Dussault *et al.*, 2004; Montgomery *et al.*, 2019). Montgomery *et al.* (2019) suggested that shifts to nocturnal activity could compensate for lower activity during the day when ambient conditions are warmer. We propose this change in moose movement rates provides an additional thermoregulatory benefit from foraging. Observed activity rates in these same moose during the summer found 75% of active movement rates were associated with foraging (Herberg, 2017). Furthermore, our study detected decreasing rates of change in rumen temperature during low to moderate movement rates in moose, which we assume are foraging bouts. When ambient conditions are cooler at night, presumably the temperature of the available vegetation on the landscape is also cooler and may become covered in condensation. Foraging on this cool, wet vegetation may allow moose to facilitate the decline in core body temperature to the daily nadir around mid-day (Thompson *et al.*, 2019).

Our study associates behavioural choices of moose with the rate of change in rumen temperature to confirm previous reports of habitat selection for behavioural thermoregulation in moose (Dussault *et al.*, 2004; Broders *et al.*, 2012; Street *et al.*, 2015; Wattles *et al.*, 2018; Alston *et al.*, 2020; Jennewein *et al.*, 2020). Open meadow habitats were associated with slightly higher rates of change in rumen temperature on tolerance days; these meadows may have been selected for resting in the early morning when ambient air temperatures were the coldest of all available habitat types (McCann *et al.*, 2016). Furthermore, selection of wetlands increased and resulted in a decrease in the rate of change rumen temperature on tolerance days, with higher selection in the late evening corresponding to higher probabilities of drinking. Resting behaviour by moose in all habitats except wetlands in this study resulted in increasing rumen temperatures. During the summer, moose spend 47–67% of their time bedded, allowing them to rest and ruminate (Renecker and Hudson, 1989; Herberg, 2017). Increases in rumen temperature of resting moose can be attributed to diet-induced thermogenesis and rumination (Lawler and White, 2003; Herberg *et al.*, 2018). We did not detect moose were selecting for specific microhabitats during tolerance days compared to control days; however, we did document moose selected features within each habitat that could provide thermal relief. In forested habitats, moose selected elevated resting sites, which could provide access to wind. Wind can decrease the physiological response of moose to warm ambient conditions (Renecker and Hudson, 1990; McCann *et al.*, 2013) and provide relief from biting insects (Mörschel and Klein, 1997). Additionally, our results show moose selected resting sites in forested habitats with denser canopy cover than the surrounding area to reduce heat loads from solar radiation (Demarchi and Bunnell, 1993; Melin *et al.*, 2014; McCann *et al.*, 2016).

Our study demonstrates behavioural choices of moose on the landscape are associated with the rate of change in rumen temperature and their ability to thermoregulate. Although rumen temperature correlates with core body temperature (Signer *et al.*, 2010; Herberg *et al.*, 2018), inherent biases in rumen temperature are present when animals ingest forage and water. Future studies could use body or vaginal implant temperature loggers to track core body temperature as a response to behaviour. Additionally, using fine-scale accelerometers, magnetometers, and GPS collars could improve behavioural classifications (Hughey *et al.*, 2018) and create dead-reckoned pathways between GPS points (Wensveen *et al.*, 2015) to determine real time habitat use. We suggest future studies on moose response to environmental conditions should incorporate the animal indicator concept (Franzmann, 1983), with body temperature as a physiological indicator of potential heat stress. Individual indices of thermal response advance the classical environmental indicator concept of lower and upper critical temperature thresholds that have been applied to all individuals in all habitats (Renecker and Hudson, 1986; Montgomery *et al.*, 2019; Weiskopf *et al.*, 2019). For large terrestrial herbivores, the animal indicator concept has provided an avenue to understand how trade-offs impact life history characteristics of wildlife populations (Monteith *et al.*, 2013; Bårdsen *et al.*, 2014; White *et al.*, 2014; Long *et al.*, 2016; Middleton *et al.*, 2018). In this study, we applied the animal indicator concept by incorporating the thermal response with continuous core body temperature measurements. Aspects of the landscape of fear (Ditmer *et al.*, 2017, 2018; Oates *et al.*, 2019), including insect harassment (Renecker and Hudson, 1990; Mörschel and Klein, 1997), could also be considered with the animal indicator concept to determine how these factors ultimately influence their life history characteristics with warming temperatures. Ultimately, these trade-offs may be limited by the availability of high-value habitats (Elmore *et al.*, 2017) that could reduce negative effects of warm environmental conditions. Wildlife managers must consider high-value habitats where wildlife can employ both behavioural and physiological mechanisms to tolerate warm ambient conditions in a landscape of forage, predators and pests.

## Funding

This work was supported by the Alaska Department of Fish and Game Federal Wildlife Restoration Grant [grant number AKW-4 Project No. 1.63]. Minnesota Department of Natural Resources provided GPS collars and mortality implant transmitters to record rumen temperature for this project. Support for analysis, writing and publication was provided by Texas A&M University and the Boone & Crockett Dr James H. ‘Red’ Duke Wildlife Conservation and Policy Program.

## Supplementary Material

suppl_data_coaa130

## References

[ref1] Alduchov OA, Eskridge RE (1996) Improved Magnus form approximation of saturation vapor pressure. J Appl Meteorol 35: 601–609.

[ref2] Alston JM, Joyce MJ, Merkle JA, Moen RA (2020) Temperature shapes movement and habitat selection by a heat-sensitive ungulate. Landsc Ecol 35: 1961–1973.

[ref3] Arnold W, Ruf T, Loe LE, Irvine RJ, Ropstad E, Veiberg V, Albon SD (2018) Circadian rhythmicity persists through the polar night and midnight sun in Svalbard reindeer. Sci Rep 8: 14466. 30262810 10.1038/s41598-018-32778-4 PMC6160466

[ref4] Ballenberghe V, Miquelle DG (1990) Activity of moose during spring and summer in Interior Alaska. J Wildl Manage 54: 391.

[ref5] Barboza PS, Parker KL, Hume ID (2009) Integrative Wildlife Nutrition. Springer, Berlin Heidelberg

[ref6] Bårdsen B-J, Næss MW, Tveraa T, Langeland K, Fauchald P (2014) Risk-sensitive reproductive allocation: fitness consequences of body mass losses in two contrasting environments. Ecol Evol 4: 1030–1038. 24772280 10.1002/ece3.1010 PMC3997319

[ref7] Baston D (2019) Fast extraction from raster datasets using polygons R Package: Version 0.1.1.

[ref8] Boyers M, Parrini F, Owen-smith N, Erasmus BFN, Hetem RS (2019) How free-ranging ungulates with differing water dependencies cope with seasonal variation in temperature and aridity. Conserv Physiol 7: doi:10.1093/conphys/coz064. 10.1093/conphys/coz064 PMC6839429 31723430

[ref9] Brain C, Mitchell D (1999) Body temperature changes in free-ranging baboons (*Papio hamadryas ursinus*) in the Namib Desert, Namibia. Int J Primatol 20: 585–598.

[ref10] Brivio F, Zurmühl M, Grignolio S, von Hardenberg J, Apollonio M, Ciuti S (2019) Forecasting the response to global warming in a heat-sensitive species. Sci Rep 9: 1–16. 30816191 10.1038/s41598-019-39450-5 PMC6395821

[ref11] Broders HG, Coombs AB, Mccarron JR (2012) Ectothermic responses of moose (*Alces alces*) to thermoregulatory stress on mainland Nova Scotia. Alces 48: 53–61.

[ref84] Burnham KP, Anderson DR (2002) Model Selection and Multimodel Inference - A Practical Information-Theoretic Approach, Second Edi. Edition. Springer-Verlag, New York, New York, USA.

[ref12] Cain JW III, Krausman PR, Rosenstock SS, Turner JC (2006) Mechanisms of thermoregulation and water balance in desert ungulates. Wildl Soc Bull 34: 570–581.

[ref13] Camp MJ, Shipley LA, Milling CR, Rachlow JL, Forbey JS (2018) Interacting effects of ambient temperature and food quality on the foraging ecology of small mammalian herbivores. J Therm Biol 71: 83–90. 29301704 10.1016/j.jtherbio.2017.10.021

[ref14] Collins WB, Schwartz CC (1998) Logging in Alaska’s boreal forest: creation of grasslands or enhancement of moost habitat. Alces 34: 355–374.

[ref15] Demarchi MW, Bunnell FL (1993) Estimating forest canopy effects on summer thermal cover for the Cervidae (deer family). Can J For Res 23: 2419–2426.

[ref16] Demarchi MW, Bunnell FL (1995) Forest cover selection and activity of cow moose in summer. Acta Theriol (Warsz) 40: 23–36.

[ref17] Diamond HJ, Karl TR, Palecki MA, Baker CB, Bell JE, Leeper RD, Easterling DR, Lawrimore JH, Meyers TP, Helfert MR, et al. (2013) U.S. climate reference network after one decade of operations status and assessment. Bull Am Meteorol Soc 94: 485–498.

[ref18] Ditmer MA, Fieberg JR, Moen RA, Windels SK, Stapleton SP, Harris TR (2018) Moose movement rates are altered by wolf presence in two ecosystems. Ecol Evol 8: 9017–9033. 30271563 10.1002/ece3.4402 PMC6157672

[ref19] Ditmer MA, Moen RA, Windels SK, Forester JD, Ness TE, Harris TR (2017) Moose at their bioclimatic edge alter their behavior based on weather, landscape, and predators. Curr Zool 4:1–14. 10.1093/cz/zox047 PMC6084617 30109872

[ref20] Dussault C, Ouellet J-P, Courtois R, Huot J, Breton L, Larochelle J (2004) Behavioural responses of moose to thermal conditions in the boreal forest. Ecoscience 11: 321–328.

[ref21] Elmore RD, Carroll JM, Tanner EP, Hovick TJ, Grisham BA, Fuhlendorf SD, Windels SK (2017) Implications of the thermal environment for terrestrial wildlife management. Wildl Soc Bull 41: 183–193.

[ref22] Fick LG, Kucio TA, Fuller A, Matthee A, Mitchell D (2009) The relative roles of the parasol-like tail and burrow shuttling in thermoregulation of free-ranging Cape ground squirrels, *Xerus inauris*. Comp Biochem Physiol A Mol Integr Physiol 152: 334–340. 19041951 10.1016/j.cbpa.2008.11.004

[ref23] Franzmann AW (1983) Health (condition) evaluation of wild animal populations: the animal indicator concept. In CC Schwartz, AW Franzmann, eds, Moose Productivity and Physiology. Alaska Department of Fish and Game, Juenau, p. 131.

[ref24] Fuller A, Kamerman PR, Maloney SK, Matthee A, Mitchell G, Mitchell D (2005) A year in the thermal life of a free-ranging herd of springbok *Antidorcas marsupialis*. J Exp Biol 208: 2855–2864. 16043590 10.1242/jeb.01714

[ref25] Gallagher AJ, Creel S, Wilson RP, Cooke SJ (2017) Energy landscapes and the landscape of fear. Trends Ecol Evol 32: 88–96. 27814919 10.1016/j.tree.2016.10.010

[ref26] Haase CG, Fletcher RJ, Slone DH, Reid JP, Butler SM (2019) Traveling to thermal refuges during stressful temperatures leads to foraging constraints in a central-place forager. J Mammal. 101:271–280. doi: 10.1093/jmammal/gyz197.

[ref27] Harrell FE (2001) Regression Modeling Strategies: With Applications to Linear Models, Logistic Regression, and Survival Analysis. Springer Science+Business Media, New York, USA.

[ref28] Herberg AM (2017) Are Minnesota moose warming up to climate change? A validation of techniques for remotely monitoring moose behavior and body temperature. MSc thesis. University of Minnesota.

[ref29] Herberg AM, St-Louis V, Carstensen M, Fieberg J, Thompson DP, Crouse JA, Forester JD (2018) Calibration of a rumen bolus to measure continuous internal body temperature in moose. Wildl Soc Bull 42: 328–337.

[ref30] Hetem RS, Maartin Strauss W, Heusinkveld BG, de Bie S, Prins HHT, van Wieren SE (2011) Energy advantages of orientation to solar radiation in three African ruminants. J Therm Biol 36: 452–460.

[ref31] Hetem RS, Strauss WM, Fick LG, Maloney SK, Meyer LCR, Shobrak M, Fuller A, Mitchell D (2012) Activity re-assignment and microclimate selection of free-living Arabian oryx: responses that could minimise the effects of climate change on homeostasis? Zoology 115: 411–416. 23036437 10.1016/j.zool.2012.04.005

[ref32] Hughey LF, Hein AM, Strandburg-Peshkin A, Jensen FH (2018) Challenges and solutions for studying collective animal behaviour in the wild. Philos Trans R Soc B Biol Sci 373: 1–13. 10.1098/rstb.2017.0005 PMC5882975 29581390

[ref33] Jennewein JS, Hebblewhit M, Gilbert S, Meddens AJH, Boelman NT, Joly K, Jones K, Kellie KA, Brainerd S, et al. (2020) Behavioral modifications by a large-northern herbivore to mitigate warming conditions. Mov Ecol 8: 1–14. 33072330 10.1186/s40462-020-00223-9 PMC7559473

[ref34] Laundré JW, Hernández L, Altendorf KB (2001) Wolves, elk, and bison: reestablishing the “landscape of fear” in Yellowstone National Park, U.S.A. Can J Zool 79: 1401–1409.

[ref35] Lawler JP, White RG (2003) Temporal responses in energy expenditure and respiratory quotient following feeding in the muskox: influence of season on energy costs of eating and standing and an endogenous heat increment. Can J Zool 81: 1524–1538.

[ref36] Lease HM, Murray IW, Fuller A, Hetem RS (2014) Black wildebeest seek shade less and use solar orientation behavior more than do blue wildebeest. J Therm Biol 45: 150–156. 25436964 10.1016/j.jtherbio.2014.08.008

[ref37] Long RA, Bowyer RT, Porter WP, Mathewson P, Monteith KL, Findholt SL, Dick BL, Kie JG (2016) Linking habitat selection to fitness-related traits in herbivores: the role of the energy landscape. Oecologia 181: 709–720. 27003702 10.1007/s00442-016-3604-7

[ref38] Long RA, Bowyer RT, Porter WP, Mathewson P, Monteith KL, Kie JG (2014) Behavior and nutritional condition buffer a large-bodied endotherm against direct and indirect effects of climate. Ecol Monogr 84: 513–532.

[ref39] Long RA, Martin TJ, Barnes BM (2005) Body temperature and activity patterns in free-living Arctic ground squirrels. J Mammal 86: 314–322.

[ref40] Lowe SJ (2009) Behavioral responses of moose (*Alces alces*) to ambient temperature: is there evidence for behavioral thermoregulation? MSc thesis. Trent University.

[ref41] Maloney SK, Moss G, Cartmell T, Mitchell D (2005a) Alteration in diel activity patterns as a thermoregulatory strategy in black wildebeest (*Connochaetes gnou*). J Comp Physiol A 191: 1055–1064. 10.1007/s00359-005-0030-4 16049700

[ref42] Maloney SK, Moss G, Mitchell D (2005b) Orientation to solar radiation in black wildebeest (*Connochaetes gnou*). J Comp Physiol A 191: 1065–1077. 10.1007/s00359-005-0031-3 16075268

[ref43] McCann NP, Moen RA, Harris TR (2013) Warm-season heat stress in moose (*Alces alces*). Can J Zool 91: 893–898.

[ref44] McCann NP, Moen RA, Windels SK, Harris TR (2016) Bed sites as thermal refuges for a cold-adapted ungulate in summer. Wildlife Biol 22: 228–237.

[ref45] McFarland R, Barrett L, Boner R, Freeman NJ, Henzi SP (2014) Behavioral flexibility of vervet monkeys in response to climatic and social variability. Am J Phys Anthropol 154: 357–364. 24706453 10.1002/ajpa.22518

[ref46] McFarland R, Barrett L, Fuller A, Hetem RS, Maloney SK, Mitchell D, Henzi SP (2019) Keeping their cool: behavioral thermoregulation and body temperature patterns of wild vervet monkeys. Am J Phys Anthropol 168: 1–283. 10.1002/ajpa.23962 31713853

[ref47] Melin M, Matala J, Mehtätalo L, Tiilikainen R, Tikkanen O-P, Maltamo M, Pusenius J, Packalen P (2014) Moose (*Alces alces*) reacts to high summer temperatures by utilizing thermal shelters in boreal forests - an analysis based on airborne laser scanning of the canopy structure at moose locations. Glob Chang Biol 20: 1115–1125. 24115403 10.1111/gcb.12405

[ref48] Middleton AD, Merkle JA, McWhirter DE, Cook JG, Cook RC, White PJ, Kauffman MJ (2018) Green-wave surfing increases fat gain in a migratory ungulate. Oikos 127: 1060–1068.

[ref49] Miner B (2000) Forest regeneration and use of browse by moose in large-scale wildfires and managed habitat areas, Kenai National Wildlife Refuge, Alaska. MSc thesis. Alaska Pacific University.

[ref50] Minicucci L, Carstensen M, Crouse J, Arnemo JM, Evans A (2018) A technique for deployment of rumen bolus transmitters in free-ranging moose (*Alces alces*). J Zoo Wildl Med 49: 227–230. 29517432 10.1638/2017-0027R.1

[ref51] Monteith KL, Stephenson TR, Bleich VC, Conner MM, Pierce BM, Bowyer RT (2013) Risk-sensitive allocation in seasonal dynamics of fat and protein reserves in a long-lived mammal. J Anim Ecol 82: 377–388. 23379674 10.1111/1365-2656.12016

[ref52] Montgomery RA, Redilla KM, Moll RJ, Van Moorter B, Rolandsen CM, Millspaugh JJ, Solberg EJ (2019) Movement modeling reveals the complex nature of the response of moose to ambient temperatures during summer. J Mammal 100: 169–177.

[ref53] Mörschel FM, Klein DR (1997) Effects of weather and parasitic insects on behavior and group dynamics of caribou of the Delta herd, Alaska. Can J Zool 75: 1659–1670.

[ref54] Oates BA, Merkle JA, Kauffman MJ, Dewey SR, Jimenez MD, Vartanian JM, Becker SA, Goheen JR (2019) Antipredator response diminishes during periods of resource deficit for a large herbivore. Ecology 100: e02618. 30865296 10.1002/ecy.2618

[ref55] Olson BT, Windels SK, Moen RA, Mccann NP (2016) Moose modify bed sites in response to high temperatures. Alces 52:153–160.

[ref56] R Core Team (2018) R: A Language and Environment for Statistical Computing. R Foundation for Statistical Computing, Vienna, Austria

[ref57] Rabe-Hesketh S, Skrondal A (2010) Multilevel and Longitudinal Modeling Using Stata. Volume I: Continuous Responses, Ed 3. Stata Press, College Station, Texas, USA.

[ref58] Renecker LA, Hudson RJ (1986) Seasonal energy expenditures and thermoregulatory responses of moose. Can J Zool 64: 322–327.

[ref59] Renecker LA, Hudson RJ (1989) Seasonal activity budgets of moose in aspen-dominated boreal forests. J Wildl Manage 53: 296–302.

[ref60] Renecker LA, Hudson RJ (1990) Behavioral and thermoregulatory responses of moose to high ambient temperatures and insect harassment in aspen-dominated forests. Alces 26: 66–72.

[ref61] Rey B, Fuller A, Hetem RS, Lease HM, Mitchell D, Meyer LCR (2016) Microchip transponder thermometry for monitoring core body temperature of antelope during capture. J Therm Biol 55: 47–53. 26724197 10.1016/j.jtherbio.2015.11.010

[ref62] Riek A, Brinkmann L, Gauly M, Perica J, Ruf T, Arnold W, Hambly C, Speakman JR, Gerken M (2017) Seasonal changes in energy expenditure, body temperature and activity patterns in llamas (*Lama glama*). Sci Rep 7: 1–12. 28790450 10.1038/s41598-017-07946-7 PMC5548813

[ref63] Schwartz CC, Regelin WL, Franzmann AW (1984) Seasonal dynamics of food intake in moose. Alces 20: 223–244.

[ref64] Shively RD, Crouse JA, Thompson DP, Barboza PS (2019) Is summer food intake a limiting factor for boreal browsers? Diet, temperature, and reproduction as drivers of consumption in female moose. PLoS One 14: e0223617. 31596894 10.1371/journal.pone.0223617 PMC6785127

[ref65] Shrestha AK, van Wieren SE, van Langevelde F, Fuller A, Hetem RS, Meyer L, de Bie S, Prins HHT (2014) Larger antelopes are sensitive to heat stress throughout all seasons but smaller antelopes only during summer in an African semi-arid environment. Int J Biometeorol 58: 41–49. 23417331 10.1007/s00484-012-0622-y

[ref66] Signer C, Ruf T, Schober F, Fluch G, Paumann T, Arnold W (2010) A versatile telemetry system for continuous measurement of heart rate, body temperature and locomotor activity in free-ranging ruminants. Methods Ecol Evol 1: 75–85. 22428081 10.1111/j.2041-210X.2009.00010.x PMC3303127

[ref67] Silanikove N (2000) Effects of heat stress on the welfare of extensively managed domestic ruminants. Livest Prod Sci 67: 1–18.

[ref68] Street GM, Rodgers AR, Fryxell JM (2015) Mid-day temperature variation influences seasonal habitat selection by moose. J Wildl Manage 79: 505–512.

[ref69] Terrien J, Perret M, Aujard F (2011) Behavioral thermoregulation in mammals: a review. Front Biosci 16: 1428–1444. 10.2741/3797 21196240

[ref70] Thaker M, Gupte PR, Prins HHT, Slotow R, Vanak AT (2019) Fine-scale tracking of ambient temperature and movement reveals shuttling behavior of elephants to water. Front Ecol Evol 7: 1–12.

[ref71] Thompson DP, Barboza PS, Crouse JA, McDonough TJ, Badajos OH, Herberg AM (2019) Body temperature patterns vary with day, season, and body condition of moose (*Alces alces*). J Mammal 100: 1466–1478.

[ref72] Thompson DP, Crouse JA, Jaques S, Barboza PS (2020) Redefining physiological responses of moose (*Alces alces*) to warm environmental conditions. J Therm Biol 90: 102581. 32479386 10.1016/j.jtherbio.2020.102581

[ref73] Thompson DP, Crouse JA, McDonough TJ, Badajos OH, Adsem J, Barboza PS (2018) Vaginal implant transmitters for continuous body temperature measurement in moose. Wildl Soc Bull 42: 321–327.

[ref74] Turbill C, Ruf T, Mang T, Arnold W (2011) Regulation of heart rate and rumen temperature in red deer: effects of season and food intake. J Exp Biol 214: 963–970. 21346124 10.1242/jeb.052282 PMC3280896

[ref75] Valeix M, Fritz H, Matsika R, Matsvimbo F, Madzikanda H (2008) The role of water abundance, thermoregulation, perceived predation risk and interference competition in water access by African herbivores. Afr J Ecol 46: 402–410.

[ref76] van Beest FM, Van Moorter B, Milner JM (2012) Temperature-mediated habitat use and selection by a heat-sensitive northern ungulate. Anim Behav 84: 723–735.

[ref77] Wattles DW, DeStefano S (2013) Moose habitat in Massachusetts: assessing use at the southern edge of the range. Alces 49: 133–147.

[ref78] Wattles DW, Zeller KA, DeStefano S (2018) Range expansion in unfavorable environments through behavioral responses to microclimatic conditions: moose (*Alces americanus*) as the model. Mamm Biol 93: 189–197.

[ref79] Weiskopf SR, Ledee OE, Thompson LM (2019) Climate change effects on deer and moose in the Midwest. J Wildl Manage 83: 769–781.

[ref80] Wensveen PJ, Thomas L, Miller PJO (2015) A path reconstruction method integrating dead-reckoning and position fixes applied to humpback whales. Mov Ecol 3: 1–16. 26392865 10.1186/s40462-015-0061-6 PMC4576411

[ref81] White KS, Barten NL, Crouse S, Crouse J (2014) Benefits of migration in relation to nutritional condition and predation risk in a partially migratory moose population. Ecology 95: 225–237. 24649661 10.1890/13-0054.1

[ref82] Wiemers DW, Fulbright TE, Wester DB, Ortega-S JA, Rasmussen GA, Hewitt DG, Hellickson MW (2014) Role of thermal environment in habitat selection by male white-tailed deer during summer in Texas, USA. Wildlife Biol 20: 47–56.

[ref83] Wilson RP, Quintana F, Hobson VJ (2012) Construction of energy landscapes can clarify the movement and distribution of foraging animals. Proc R Soc B Biol Sci 279: 975–980. 10.1098/rspb.2011.1544 PMC3259934 21900327

