# Supplementary material for: Behaviour influences thermoregulation of boreal moose during the warm season
Source: Conserv Physiol. 2021 Jan 8;9(1):coaa130. doi: 10.1093/conphys/coaa130 (PMC7799588; doi:10.1093/conphys/coaa130)

**Supplementary Data**

**Title:** Behavior influences thermoregulation in boreal moose during the warm season

**Journal:** Conservation Physiology

**Authors:** Daniel P. Thompson, John. A. Crouse, Perry S. Barboza, Miles O. Spathelf, Andrew M. Herberg, Stephanie D. Parker, Max A. Morris.

**Corresponding Author:** Daniel P. Thompson, Alaska Department of Fish and Game, Kenai Moose Research Center, 43961 Kalifornsky Beach Road Suite B, Soldotna, AK 99669, USA. email: dan.thompson2@alaska.gov; telephone: 907-260-2953

**Supplementary Data S1**. Defining habitats within the Kenai Moose Research Center, Alaska, USA.

Tree density was evaluated by counting the number of trees in two subplots (3.56 m or 8.93 m radius) placed 36.5 m apart (Herrick, J. E., J. W. Van Zee, K. M. Havstad, L. M. Burkett, and W. G. Whitford. 2005. Monitoring manual for grassland, shrubland, and savanna ecosystems. Volume II: Design, supplementary methods and interpretation. USDA-ARS Jornada Experimental Range, Las Cruces, NM, USA). The size of each tree within the radius was measured by diameter at breast height (cm; DBH) and estimated height (m). Understory vegetation was noted, but not measured in a repeatable fashion.

Early seral boreal forest consisted of high densities of fireweed (*Epilobium angustifolium*), prickly rose (*Rosa acicularis*), Nootka lupine (*Lupinus nootkatensis*), and seedlings (<1m tall) of quaking aspen (*Populus tremuloides*), paper birch (*Betula papyrifera*), and white spruce (*Picea glauca*). Mid seral boreal forest contained a mixture of quaking aspen, paper birch, Scouler’s willow (*Salix scouleriana*) and white spruce (stems • ha^-1^ = 6,700 ± 2,800 SD; DBH = 6.0 cm ± 1.6 cm; height 8.1 m ± 2.0 m SD), with a medium density understory of lowbush cranberry (*Vaccinium vitus-idaea*), dwarf dogwood (*Cornus canadensis*), ferns (*Athyrium filix-femina; Gymnocarpium dryopteris*), fireweed and prickly rose. Late seral boreal forest consisted of a mixture of quaking aspen, paper birch and white spruce (stems • ha^-1^ = 1,400 ± 400 SD; DBH = 15.0 cm ± 3.0 cm; height 11.3 m ± 2.5 m SD). Late seral boreal forest had a low density understory of feather moss (*Pleurozium schreberi; Hylocomium splendens*), lowbush cranberry, highbush cranberry (*Viburnum edule*), and ferns, with pockets of devil’s club (*Oplopanax horridus*) and false azalea (*Menziesta ferruginea*). Black spruce (*Picea mariana*) forest (stems • ha^-1^ = 8,500 ± 2,900 SD; DBH = 6.1 cm ± 1.5 cm; height 7.5 m ± 2.1 m SD) had a very low density of understory which consisted predominantly of feather and sphagnum moss (*Sphagnum* spp.), lowbush cranberry, and sporadic fireweed and prickly rose. Wetlands consisted of sphagnum moss, sedges (*Carex* spp.), bog rosemary (*Andromeda polifolia*), sweet gale (*Myrica gale*), and Labrador tea (*Ledum groenlandicum*), with intermittent stunted black spruce along the edges. Graminoids (*Festuca altaica*; *Calamagrostis canadensis*), fireweed, and Nootka lupine were the dominant vegetation in open meadows, which were intermixed with sporadic white spruce and pockets of dwarf birch (*Betula glandulosa*).

**Supplementary Data S2**. Environmental conditions and moose tolerance days during May through August 2015 at the Kenai Moose Research Center on the Kenai Peninsula, Alaska, USA. (A) Daily range in ambient air temperature (°C), (B) daily range in vapor pressure (hPa), (C) daily range in solar radiation (W • m^-2^), (D) total daily precipitation (mm), and (E) mean daily wind speed (m • s^-1^) recorded by a National Oceanic and Atmospheric Administration, U.S. Climate Reference Network (AK Kenai 29 ENE) weather station. (F) Frequency of moose tolerance days when individual moose had a daily amplitude in body temperature ≥1.2°C recorded in captive adult female moose (*n* = 6).


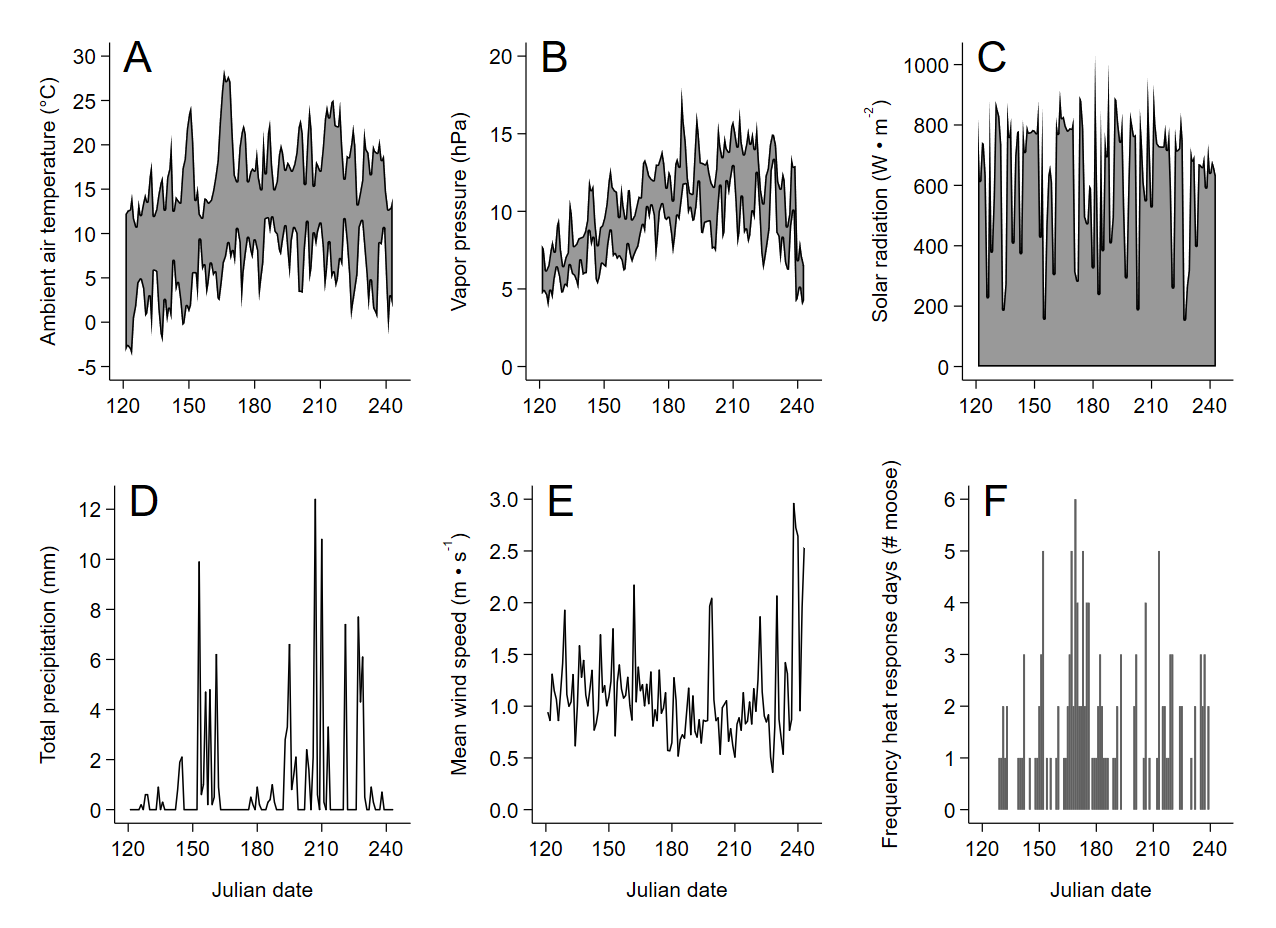


**Supplementary Data S3**. Models for thermal and behavioral responses to hot days in captive adult female moose (*n* = 6) at the Kenai Moose Research Center on the Kenai Peninsula, Alaska, USA during May through August of 2015.

Ranking of mixed-effects regression models for three dependent variables: linear model for the rate of change in rumen temperature (°C • h^-1^), linear model of movement rate (m • h^-1^), and logistic model of the probability of drinking. Model selection used Akaike’s information criterion (AIC*_c_*; corrected for small sample size). ΔAIC*_c_* = difference between model AIC*_c_* and lowest AIC*_c_* in the model set. ω = Akaike model weight. *k* = number of estimable parameters. Deviance = measure of model fit. Model covariates for all 3 dependent variables included the categorical variable for daily thermoregulatory response (DTR; tolerance days when individual moose had a daily amplitude in body temperature ≥1.2°C; control as 1 day prior/after a tolerance day), the categorical variable for time of day (TOD; 0.5-h time periods starting at 0:00), and the interaction between daily thermoregulatory response and time of day (DTR:TOD). All models include 2 additional parameters for individual as a random effect and the associated error term. * Best model determined by selecting the simplest model with the lowest AICc within 2 AICc of the top model.

| no. | Model | ΔAIC*_c_* | ω | *k* | Deviance |
| --- | --- | --- | --- | --- | --- |
| Change in rumen temperature (°C • h^-1^) | |  |  |  |  |
| 1* | TOD | 0.00 | 0.72 | 50 | -11528.73 |
| 2 | DTR + TOD | 1.91 | 0.28 | 51 | -11528.84 |
| 3 | DTR:TOD | 10.20 | 0.00 | 98 | -11615.48 |
| 4 | null | 177.48 | 0.00 | 3 | -11256.92 |
| 5 | DTR | 179.38 | 0.00 | 4 | -11257.02 |
|  |  |  |  |  |  |
| Movement rate (m • h^-1^) | |  |  |  |  |
| 1* | DTR + TOD | 0.00 | 0.87 | 51 | 190273.48 |
| 2 | TOD | 17.53 | 0.00 | 50 | 190295.04 |
| 3 | DTR:TOD | 125.14 | 0.00 | 98 | 190204.04 |
| 4 | DTR | 1180.02 | 0.00 | 4 | 191549.88 |
| 5 | null | 1195.86 | 0.00 | 3 | 191569.72 |
|  |  |  |  |  |  |
| Probability of drinking | |  |  |  |  |
| 1* | TOD | 0.00 | 0.68 | 48 | 5852.77 |
| 2 | DTR + TOD | 1.51 | 0.32 | 49 | 5850.25 |
| 3 | DTR:TOD | 162.72 | 0.00 | 92 | 5816.75 |
| 4 | null | 243.02 | 0.00 | 2 | 6192.13 |
| 5 | DTR | 244.56 | 0.00 | 3 | 6189.67 |

**Supplementary Data S4**. Models for rate of change rumen temperature influenced by habitat type, movement, and daily thermoregulatory response in adult female moose.

Ranking of linear mixed-effect model regression for the rate of change in rumen temperature (°C • h^-1^) recorded in captive adult female moose (*n* = 6) at the Kenai Moose Research Center on the Kenai Peninsula, Alaska during May through August of 2015. Model selection used Akaike’s information criterion (AIC*_c_*; corrected for small sample size). ΔAIC*_c_* = difference between model AIC*_c_* and lowest AIC*_c_* in the model set. ω = Akaike model weight. *k* = number of estimable parameters. Deviance = measure of model fit. Model covariates included the categorical variable for daily thermoregulatory response (DTR; tolerance days when individual moose had a daily amplitude in body temperature ≥1.2°C; control as 1 day prior/after a tolerance day), the categorical variable for habitat type (HT; 1-6), a spline fit to the continuous variable movement rate (MR; 7 knots based on percentiles for large sample sizes (Harrell 2001)), and the two-way interaction between the categorical variables (DTR:HT). All models include 2 additional parameters for individual as a random effect and the associated error term. All models with movement rate include 6 additional parameters for the spline. *Best model determined by selecting the simplest model with the lowest AICc within 2 AICc of the top model.

| no. | Model | ΔAIC_c_ | ω | *k* | Deviance |
| --- | --- | --- | --- | --- | --- |
| 1* | HT + DTR + ED + DTR:HT | 0.00 | 1.00 | 20 | -12634.00 |
| 2 | HT + DTR + ED | 21.29 | 0.00 | 14 | -12600.68 |
| 3 | DTR + ED | 23.28 | 0.00 | 15 | -12600.70 |
| 4 | HT + ED | 79.60 | 0.00 | 9 | -12532.36 |
| 5 | HT + DTR | 81.59 | 0.00 | 10 | -12532.36 |
| 6 | HT | 1263.46 | 0.00 | 8 | -11346.49 |
| 7 | DTR | 1265.44 | 0.00 | 9 | -11346.52 |
| 8 | ED | 1343.03 | 0.00 | 3 | -11256.92 |
| 9 | null | 1344.92 | 0.00 | 4 | -11257.02 |

**Supplementary Data S5**. Rate of change in rumen temperature (°C • h^-1^) by habitat type and daily thermoregulatory response collected from captive adult female moose (*n* = 6) at the Kenai Moose Research Center, Kenai Peninsula, Alaska, USA from May through August 2015. Tolerance days were those days when individual moose had a daily amplitude in body temperature ≥1.2°C. Control days were the day prior and the day after a tolerance day. Habitat types include open meadow (OM), black spruce forest (BSF), early seral boreal forest (ESBF), mid seral boreal forest (MSBF), late seral boreal forest (LSBF), and wetlands (WL). Predicted values with 95% confidence intervals from mixed-effect model regression.


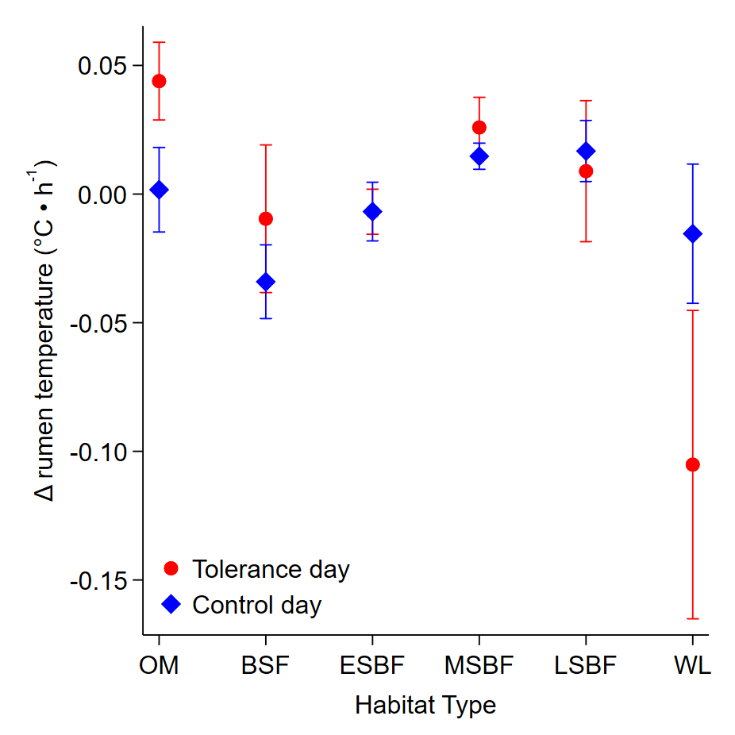


**Supplementary Data S6**. Models for the probability of adult moose selecting habitats influenced by daily thermoregulatory response, activity status, and time of day.

Ranking of logistic model regression for the probability of selecting 6 different habitats by captive adult female moose (*n* = 6) at the Kenai Moose Research Center on the Kenai Peninsula, Alaska during May through August of 2015. Model selection used Akaike’s information criterion (AIC*_c_*; corrected for small sample size). ΔAIC*_c_* = difference between model AIC*_c_* and lowest AIC*_c_* in the model set. ω = Akaike model weight. *k* = number of estimable parameters. Deviance = measure of model fit. Model covariates included the categorical variable for daily thermoregulatory response (DTR; tolerance days when individual moose had a daily amplitude in body temperature ≥1.25°C; control as 1 day prior/after a tolerance day), the categorical variable for activity status (AS; active, resting), the categorical variable for time of day (TOD; 0.5-h time periods starting at 0:00), and all two way and the three way interactions of the independent variables (DTR:AS; DTR:TOD, AS:TOD, DTR:AS:TOD). All models include 2 additional parameters for individual as a random effect and the associated error term. Models reported have sum of model weights < 0.95. * Best model determined by selecting the simplest model with the lowest AICc within 2 AICc of the top model.

| no. | Model | ΔAIC_c_ | ω | *k* | Deviance |
| --- | --- | --- | --- | --- | --- |
| Open Meadow | |  |  |  |  |
| 1* | AS + TOD | 0.00 | 0.86 | 50 | 10910.70 |
| 2 | DTR + AS + TOD | 3.96 | 0.12 | 51 | 10910.63 |
|  |  |  |  |  |  |
| Black Spruce Forest | |  |  |  |  |
| 1* | DTR + AS + TOD | 0.00 | 0.94 | 51 | 5611.07 |
| 2 | AS + TOD | 6.75 | 0.03 | 50 | 5621.85 |
|  |  |  |  |  |  |
| Early Seral Forest | |  |  |  |  |
| 1* | DTR + AS + TOD + AS:TOD | 0.00 | 0.72 | 98 | 15984.25 |
| 2 | DTR + AS + TOD + AS:DTR + AS:TOD | 1.93 | 0.28 | 99 | 15978.02 |
|  |  |  |  |  |  |
| Mid Seral Forest | |  |  |  |  |
| 1 | DTR + AS + TOD | 0.00 | 0.63 | 50 | 12700.62 |
| 2* | AS + TOD | 1.09 | 0.36 | 51 | 12697.68 |
|  |  |  |  |  |  |
| Late Seral Forest | |  |  |  |  |
| 1* | AS + TOD | 0.00 | 0.87 | 50 | 12909.69 |
| 2 | DTR + AS + TOD | 3.80 | 0.13 | 51 | 12909.46 |
|  |  |  |  |  |  |
| Wetland | |  |  |  |  |
| 1* | DTR + AS + TOD | 0.00 | 0.94 | 51 | 7110.62 |
| 2 | DTR + AS + TOD + DTR:AS | 5.87 | 0.05 | 52 | 7108.43 |

**Supplementary Data S7**. Models for microhabitat parameters for resting locations of female moose.

Ranking of linear mixed-effect model regression for elevation and canopy cover (5 m and 25 m radius) of resting locations of captive adult female moose (*n* = 6) at the Kenai Moose Research Center on the Kenai Peninsula, Alaska during May through August of 2015. Model selection used Akaike’s information criterion (AIC*_c_*; corrected for small sample size). ΔAIC*_c_* = difference between model AIC*_c_* and lowest AIC*_c_* in the model set. ω = Akaike model weight. *k* = number of estimable parameters. Deviance = measure of model fit. Model covariates for all 3 dependent variables included the categorical variable for daily thermoregulatory response (DTR; tolerance days when individual moose had a daily amplitude in body temperature ≥1.25°C; control as 1 day prior/after a tolerance day), the categorical variable for habitat type (HT; 1-6; canopy cover models only use 3 habitat types with tree canopy), and the interaction between daily thermoregulatory response and habitat type (DTR:HT). All models include 2 additional parameters for individual as a random effect and the associated error term. * Best model determined by selecting the simplest model with the lowest AICc within 2 AICc of the top model.

| no. | Model | ΔAIC_c_ | ω | *k* | Deviance |
| --- | --- | --- | --- | --- | --- |
| Elevation of rest sites | |  |  |  |  |
| 1* | HT | 0.00 | 0.69 | 8 | 5436.12 |
| 2 | DTR + HT | 1.57 | 0.31 | 9 | 5433.68 |
| 3 | DTR:HT | 13.96 | 0.00 | 14 | 5421.93 |
| 4 | null | 292.66 | 0.00 | 3 | 5740.80 |
| 5 | DTR | 294.62 | 0.00 | 4 | 5738.75 |
|  |  |  |  |  |  |
| Canopy cover in 5 m radius around rest sites | |  |  |  |  |
| 1* | HT | 0.00 | 0.72 | 5 | 30049.85 |
| 2 | DTR + HT | 2.26 | 0.23 | 6 | 30048.09 |
| 3 | DTR:HT | 5.49 | 0.05 | 8 | 30039.25 |
| 4 | null | 1032.26 | 0.00 | 3 | 31088.12 |
| 5 | DTR | 1035.65 | 0.00 | 4 | 31087.51 |
|  |  |  |  |  |  |
| Canopy cover in 25 m radius around rest sites | |  |  |  |  |
| 1* | HT | 0.00 | 0.73 | 5 | 28976.39 |
| 2 | DTR:HT | 3.04 | 0.16 | 8 | 28963.33 |
| 3 | DTR + HT | 3.83 | 0.11 | 6 | 28976.20 |
| 4 | null | 815.26 | 0.00 | 3 | 29797.67 |
| 5 | DTR | 818.67 | 0.00 | 4 | 29797.06 |

**Supplementary Data S8**. Change in elevation (A) and percent canopy cover (B) surrounding resting site locations (movement rate ≤15 m • 0.5 h^-1^) in adult female moose (n = 6). Resting sites identified from GPS collars on captive moose at the Kenai Moose Research Center on the Kenai Peninsula, Alaska, USA from May through August 2015. Habitat types include open meadow (OM), black spruce forest (BSF), early seral boreal forest (ESBF), mid seral boreal forest (MSBF), late seral boreal forest (LSBF), and wetlands (WL). Predicted values with 95% confidence intervals from mixed-effect model regression.


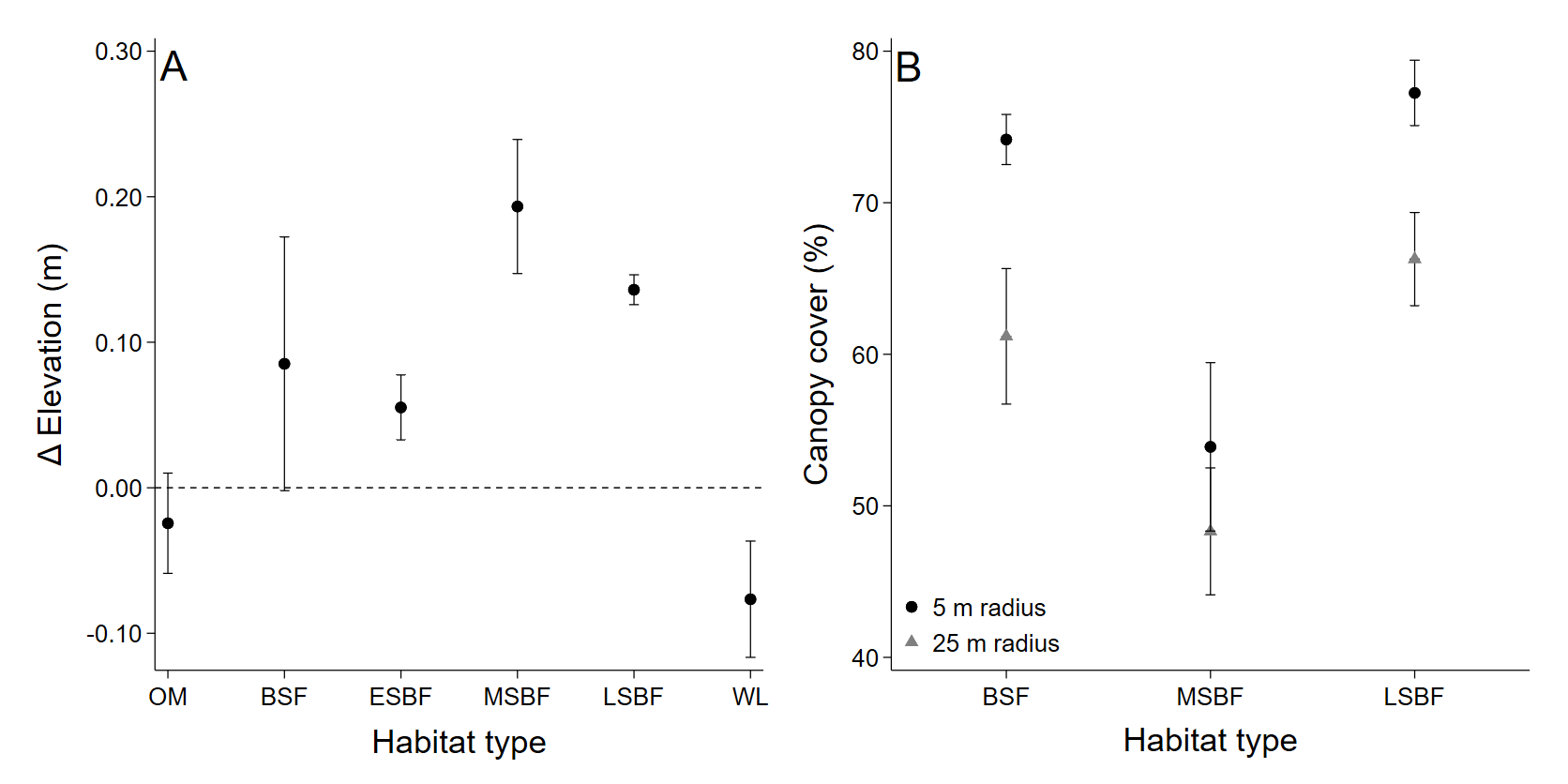

Supplement: suppl_data_coaa130 [file suppl_data_coaa130.zip › CONPHYS-2020-143_R.1_ESM_Behavior influences moose thermoregulation.docx]
